# Supplementary material for: Prognosis of Acute Ischaemic Stroke Patients with Cancer: A National Inpatient Sample Study
Source: Cancers (Basel). 2021 May 3;13(9):2193. doi: 10.3390/cancers13092193 (PMC8125286; doi:10.3390/cancers13092193)
Supplement: Supplementary file 1 [file cancers-13-02193-s001.zip › cancers-1167647-supplementary.pdf]

cancers

MDPI

Supplementary Materials

Table S1. Clinical Classifications Software Refined (CCSR) codes used to identify and classify cancer types.

Cancer Type	CCSR code	CCSR description
Head and neck cancers	NEO001	Head and neck cancers - eye
	NEO002	Head and neck cancers - lip and oral cavity
	NEO003	Head and neck cancers - throat
	NEO004	Head and neck cancers - salivary gland
	NEO005	Head and neck cancers - nasopharyngeal
	NEO006	Head and neck cancers - hypopharyngeal
	NEO007	Head and neck cancers - pharyngeal
	NEO008	Head and neck cancers - laryngeal
	NEO009	Head and neck cancers - tonsils
	NEO010	Head and neck cancers - all other types
Cardiac cancers	NEO011	Cardiac cancers
Gastrointestinal cancers - other	NEO012	Gastrointestinal cancers - esophagus
	NEO013	Gastrointestinal cancers - stomach
	NEO014	Gastrointestinal cancers - small intestine
	NEO016	Gastrointestinal cancers - anus
	NEO017	Gastrointestinal cancers - liver
	NEO018	Gastrointestinal cancers - bile duct
	NEO019	Gastrointestinal cancers - gallbladder
	NEO020	Gastrointestinal cancers - peritoneum
	NEO021	Gastrointestinal cancers - all other types
Gastrointestinal cancers - colorectal	NEO015	Gastrointestinal cancers - colorectal
Respiratory cancers	NEO022	Respiratory cancers
Bone cancer	NEO023	Bone cancer
Sarcoma	NEO024	Sarcoma
Skin cancers - melanoma	NEO025	Skin cancers - melanoma
Skin cancers - nonmelanoma	NEO026	Skin cancers - basal cell carcinoma
	NEO027	Skin cancers - squamous cell carcinoma
	NEO028	Skin cancers - all other types
Breast cancer	NEO029	Breast cancer - ductal carcinoma in situ (DCIS)
	NEO030	Breast cancer - all other types
Female reproductive system cancers	NEO031	Female reproductive system cancers - uterus
	NEO032	Female reproductive system cancers - cervix
	NEO033	Female reproductive system cancers - ovary
	NEO034	Female reproductive system cancers - fallopian tube
	NEO035	Female reproductive system cancers - endometrium
	NEO036	Female reproductive system cancers - vulva
	NEO037	Female reproductive system cancers - vagina
	NEO038	Female reproductive system cancers - all other types
Male reproductive system cancers	NEO039	Male reproductive system cancers - prostate
	NEO040	Male reproductive system cancers - testis
	NEO041	Male reproductive system cancers - penis
	NEO042	Male reproductive system cancers - all other types
Urinary system cancers	NEO043	Urinary system cancers - bladder
	NEO044	Urinary system cancers - ureter and renal pelvis
	NEO045	Urinary system cancers - kidney
	NEO046	Urinary system cancers - urethra
	NEO047	Urinary system cancers - all other types
Nervous system cancers	NEO048	Nervous system cancers - brain
	NEO049	Nervous system cancers - all other types
Endocrine system cancers	NEO050	Endocrine system cancers - thyroid

	NEO051	Endocrine system cancers - pancreas
	NEO052	Endocrine system cancers - thymus
	NEO053	Endocrine system cancers - adrenocortical
	NEO054	Endocrine system cancers - parathyroid
	NEO055	Endocrine system cancers - pituitary gland
	NEO056	Endocrine system cancers - all other types
Lymphoma	NEO057	Hodgkin lymphoma
	NEO058	Non-Hodgkin lymphoma
Leukemia	NEO059	Leukemia - acute lymphoblastic leukemia (ALL)
	NEO060	Leukemia - acute myeloid leukemia (AML)
	NEO061	Leukemia - chronic lymphocytic leukemia (CLL)
	NEO062	Leukemia - chronic myeloid leukemia (CML)
	NEO063	Leukemia - hairy cell
	NEO064	Leukemia - all other types
Multiple myeloma	NEO065	Multiple myeloma
Malignant neuroendocrine tumors	NEO066	Malignant neuroendocrine tumors
Mesothelioma	NEO067	Mesothelioma
Myelodysplastic syndrome (MDS)	NEO068	Myelodysplastic syndrome (MDS)
Cancer of other sites	NEO069	Cancer of other sites
Secondary malignancies	NEO070	Secondary malignancies
Malignant neoplasm, unspecified	NEO071	Malignant neoplasm, unspecified
Neoplasms of unspecified nature or uncertain behavior	NEO072	Neoplasms of unspecified nature or uncertain behavior
Benign neoplasms	NEO073	Benign neoplasms
Conditions due to neoplasm or the treatment of neoplasm	NEO074	Conditions due to neoplasm or the treatment of neoplasm

Table S2. International Classification of Disease – tenth edition (ICD-10) and Clinical Classifications Software Refined (CCSR) codes used to extract admission co-morbidities and procedures.

**Co-morbidities**	**ICD-10 codes (Diagnosis)**
Chronic Lung Disease	J43.x – J47.x; J60.x – J67.x; J68.4; J68.8; J68.9; J84.0x; J84.111 – J84.113; J84.115 – J84.17; J84x
Coronary Heart Disease	I20.x – I25.x
Deep Venous Thrombosis	I82.x
Pulmonary Embolism	I26.x
Pericarditis	I30.x – I32
Infectious Endocarditis	I33.0; I33.9
Atrial Fibrillation/Flutter	I48.x
Pneumonia	J12.x – J18.x
All-cause bleeding	D69.8; D69.9; G97.x; H11.3x; H31.3x; H35.6x; H43.1x; H92.2x; I31.2; I60.x—I62.x; I85.01; I85.11; K25.x-K29.x; K31.811; K62.5; K92.0-K92.2 I97.418; I97.42; I97.618-I97.621; J60.x-J62.x; N93.8; N93.9; N95.0; R04.x; R31.0; R31.9; R58; S06.4x – S06.6x
Shock	R57.x
Previous Cerebrovascular Disease	Z86.73
Family History of Stroke	Z82.3
Family History of Coronary Heart Disease	Z82.41; Z82.49
Smoking	F17.2x
Dyslipidaemia	E78.0-E78.6
**Procedures**	**ICD-10 codes (Procedural)**
Thrombolysis	03CG3ZZ; 03CG4ZZ; 03CK3Z7; 03CK3ZZ; 03CK4ZZ; 03CL3Z7; 03CL3ZZ; 03CL4ZZ; 03CP3Z7; 03CP3ZZ; 03CP4ZZ; 03CQ3Z7; 03CQ3ZZ; 03CQ4ZZ
Thrombectomy	03CG3ZZ; 03CG4ZZ; 03CK3Z7

ICD-10 - International Classification of Disease – tenth edition.

Table S3. Patient characteristics on admission, stratified by either co-existent non-metastatic or metastatic cancer. Statistically significant *p* values (<0.05) are highlighted in **bold**.

	**All**	**No cancer**	**Non-metastatic Cancer**	**Metastatic Cancer**	***p*- value**
n	1106045	1067190	20895	17960	
Age (years), median (IQR)	72 (61–82)	71 (60–82)	75 (67–83)	70 (62–78)	**<0.001**
Length-of-stay (days), median (IQR)	3 (2–6)	3 (2–6)	4 (2–7)	4 (2–7)	**<0.001**
Sex Female, *n* (%)	557595 (50.41)	538635 (50.47)	9795 (46.88)	9165 (51.03)	**<0.001**
**Ethnicity**					**<0.001**
White	735330 (66.48)	707550 (66.30)	15005 (71.81)	12775 (71.13)	
Black	183090 (16.55)	177790 (16.66)	2880 (13.78)	2420 (13.47)	
Hispanic	84950 (7.68)	82700 (7.75)	1240 (5.93)	1010 (5.62)	
Asian or Pacific Islander	31635 (2.86)	30500 (2.86)	530 (2.54)	605 (3.37)	
Native American	4700 (0.42)	4570 (0.43)	60 (0.29)	70 (0.39)	
Other	27460 (2.48)	26535 (2.49)	410 (1.96)	515 (2.87)	
**ELIXHAUSER CO-MORBIDITIES,** *n* (%)					
Congestive Heart Failure	172170 (15.57)	166950 (15.64)	3225 (15.43)	1995 (11.11)	**<0.001**
Valvular Disease	110540 (9.99)	106640 (9.99)	2300 (11.01)	1600 (8.91)	**0.008**
Pulmonary Circulation Disease	8460 (0.76)	6710 (0.63)	555 (2.66)	1195 (6.65)	**<0.001**
Peripheral Vascular Disease	112065 (10.13)	107955 (10.12)	2470 (11.82)	1640 (9.13)	**<0.001**
Paralysis	112895 (10.21)	108920 (10.21)	2260 (10.82)	1715 (9.55)	0.178
Other Neurological Disorders	6620 (0.60)	6275 (0.59)	210 (1.01)	135 (0.75)	**0.001**
Chronic Pulmonary Disease	174180 (15.75)	165690 (15.53)	4795 (22.95)	3695 (20.57)	**<0.001**
Diabetes (without chronic complications)	210220 (19.01)	203765 (19.09)	3520 (16.85)	2935 (16.34)	**<0.001**
Diabetes (with chronic complications)	214400 (19.38)	209135 (19.60)	3095 (14.81)	2170 (12.08)	**<0.001**
Hypothyroidism	159160 (14.39)	153810 (14.41)	2950 (14.12)	2400 (13.36)	0.193
Renal Failure	181950 (16.45)	175960 (16.49)	3670 (17.56)	2320 (12.92)	**<0.001**
Liver Disease	18310 (1.66)	17275 (1.62)	550 (2.63)	485 (2.70)	**<0.001**
Peptic Ulcer Disease	7695 (0.70)	7400 (0.69)	175 (0.84)	120 (0.67)	0.527
Acquired Immune Deficiency Syndrome	2395 (0.22)	2280 (0.21)	100 (0.48)	15 (0.08)	**<0.001**
Lymphoma	5315 (0.48)	4915 (0.46)	220 (1.05)	180 (1.00)	**<0.001**
Rheumatoid Arthritis / Collagen Vascular Disease	30150 (2.73)	29240 (2.74)	520 (2.49)	390 (2.17)	0.075
Coagulopathy	41405 (3.74)	37105 (3.48)	1560 (7.47)	2740 (15.26)	**<0.001**
Obesity	145465 (13.15)	142575 (13.36)	1775 (8.49)	1115 (6.21)	**<0.001**
Weight loss	44030 (3.98)	39685 (3.72)	1795 (8.59)	2550 (14.20)	**<0.001**
Fluid and electrolyte disorders	246680 (22.30)	235750 (22.09)	5200 (24.89)	5730 (31.90)	**<0.001**
Anaemia (chronic blood loss)	4025 (0.36)	3590 (0.34)	240 (1.15)	195 (1.09)	**<0.001**
Anaemia (deficiency)	133005 (12.03)	123720 (11.59)	4375 (20.94)	4910 (27.34)	**<0.001**
Alcohol abuse	49375 (4.46)	48080 (4.51)	800 (3.83)	495 (2.76)	**<0.001**
Drug abuse	28985 (2.62)	28365 (2.66)	355 (1.70)	265 (1.48)	**<0.001**
Psychoses	26255 (2.37)	25490 (2.39)	440 (2.11)	325 (1.81)	**0.041**
Depression	124635 (11.27)	120280 (11.27)	2330 (11.15)	2025 (11.28)	0.971
Hypertension	946140 (85.54)	916430 (85.87)	16975 (81.24)	12735 (70.91)	**<0.001**
**OTHER CO-MORBIDITIES** *n* (%)					
Sepsis	16400 (1.48)	15275 (1.43)	480 (2.30)	645 (3.59)	**<0.001**
Liver Disease	25520 (2.31)	24165 (2.26)	705 (3.37)	650 (3.62)	**<0.001**
Anaemia	151005 (13.65)	138965 (13.02)	5505 (26.35)	6535 (36.39)	**<0.001**

Dyslipidaemia	640010 (57.86)	621485 (58.24)	10800 (51.69)	7725 (43.01)	**<0.001**
Dementia	131650 (11.90)	128305 (12.02)	2365 (11.32)	980 (5.46)	**<0.001**
Smoking	203440 (18.39)	197625 (18.52)	3200 (15.31)	2615 (14.56)	**<0.001**
Parkinson Disease	15795 (1.43)	15335 (1.44)	315 (1.51)	145 (0.81)	**0.006**
Transient Ischaemic Attack	8070 (0.73)	7835 (0.73)	135 (0.65)	100 (0.56)	0.394
Rheumatic heart Disease	32355 (2.93)	31205 (2.92)	735 (3.52)	415 (2.31)	**0.006**
Coronary Heart Disease	314870 (28.47)	303765 (28.46)	6265 (29.98)	4840 (26.95)	**0.013**
Heart Failure	195835 (17.71)	189915 (17.80)	3600 (17.23)	2320 (12.92)	**<0.001**
Pulmonary Embolism	7000 (0.63)	5390 (0.51)	510 (2.44)	1100 (6.12)	**<0.001**
Congenital Heart Disease	33495 (3.03)	32350 (3.03)	545 (2.61)	600 (3.34)	0.169
Pericarditis	105 (0.01)	90 (0.01)	<11*	<11*	**0.018**
Infectious Endocarditis	2310 (0.21)	2050 (0.19)	70 (0.34)	190 (1.06)	**<0.001**
Atrial Fibrillation	282175 (25.51)	272015 (25.49)	6315 (30.22)	3845 (21.41)	**<0.001**
Arrhythmias (other than Atrial Fibrillation)	91590 (8.28)	88440 (8.29)	1925 (9.21)	1225 (6.82)	**0.001**
Peripheral Vascular Disease	117895 (10.66)	113575 (10.64)	2575 (12.32)	1745 (9.72)	**<0.001**
Deep Venous Thrombosis	14845 (1.34)	12395 (1.16)	840 (4.02)	1610 (8.96)	**<0.001**
Gastrointestinal Bleeding	12675 (1.15)	11600 (1.09)	560 (2.68)	515 (2.87)	**<0.001**
Pneumonia	29905 (2.70)	27575 (2.58)	1040 (4.98)	1290 (7.18)	**<0.001**
Chronic Lung Disease	136505 (12.34)	129015 (12.09)	4220 (20.20)	3270 (18.21)	**<0.001**
Chronic Obstructive Pulmonary Disease	126505 (11.44)	119750 (11.22)	3810 (18.23)	2945 (16.40)	**<0.001**
Ventricular Septal Defect	335 (0.03)	320 (0.03)	<11*	<11*	0.802
Atrial Septal Defect	31295 (2.83)	30205 (2.83)	510 (2.44)	580 (3.23)	0.122
Shock	5070 (0.46)	4830 (0.45)	95 (0.45)	145 (0.81)	**0.007**
Family history of cerebrovascular disease	42725 (3.86)	41775 (3.91)	585 (2.80)	365 (2.03)	**<0.001**
Family history of heart disease	64000 (5.79)	62450 (5.85)	840 (4.02)	710 (3.95)	**<0.001**
Previous cerebrovascular disease	172600 (15.61)	167510 (15.70)	2920 (13.97)	2170 (12.08)	**<0.001**
**PROCEDURES** *n* (%)					
Thrombectomy	34420 (3.11)	33090 (3.10)	670 (3.21)	660 (3.67)	0.139
Thrombolysis	103600 (9.37)	101035 (9.47)	1730 (8.28)	835 (4.65)	**<0.001**
**OUTCOMES**, *n* (%)					
In-hospital mortality	43545 (3.94)	40545 (3.80)	1230 (5.89)	1770 (9.86)	**<0.001**
Length-of-stay >4 days	380605 (34.41)	363430 (34.05)	8680 (41.54)	8495 (47.30)	**<0.001**
Routine Discharge	394105 (35.99)	384490 (36.39)	5600 (26.94)	4015 (22.52)	**<0.001**
**OTHER CHARACTERISTICS**, *n* (%)					
**Year of admission**					0.755
2015	112000 (10.13)	107955 (10.12)	2115 (10.12)	1930 (10.75)	
2016	488005 (44.12)	471070 (44.14)	9135 (43.72)	7800 (43.43)	
2017	506040 (45.75)	488165 (45.74)	9645 (46.16)	8230 (45.82)	
**Hospital bedsize**					**<0.001**
Small	174745 (15.80)	169030 (15.84)	3205 (15.34)	2510 (13.98)	
Medium	324895 (29.37)	314100 (29.43)	5955 (28.50)	4840 (26.95)	
Large	606405 (54.83)	584060 (54.73)	11735 (56.16)	10610 (59.08)	
**Location/teaching status of hospital**					**<0.001**
Rural	82535 (7.46)	80035 (7.50)	1505 (7.20)	995 (5.54)	
Urban non-teaching	266440 (24.09)	258170 (24.19)	4625 (22.13)	3645 (20.30)	
Urban teaching	757070 (68.45)	728985 (68.31)	14765 (70.66)	13320 (74.16)	
**Region of hospital**					**<0.001**
Northeast	198830 (17.98)	190840 (17.88)	4235 (20.27)	3755 (20.91)	
Midwest	238555 (21.57)	229900 (21.54)	4660 (22.30)	3995 (22.24)	
South	461280 (41.71)	446670 (41.85)	7945 (38.02)	6665 (37.11)	

West	207380 (18.75)	199780 (18.72)	4055 (19.41)	3545 (19.74)	
**All Patient Refined DRG: Severity of Illness Subclass**					**<0.001**
Minor loss of function	89400 (8.08)	88465 (8.29)	935 (4.47)	<11*	
Moderate loss of function	541350 (48.94)	529495 (49.62)	8025 (38.41)	3830 (21.33)	
Major loss of function	365580 (33.05)	346570 (32.48)	8955 (42.86)	10055 (55.99)	
Extreme loss of function	109715 (9.92)	102660 (9.62)	2980 (14.26)	4075 (22.69)	
**Disposition of the patient at discharge**					**<0.001**
Routine Discharge	394105 (35.63)	384490 (36.03)	5600 (26.80)	4015 (22.36)	
Transfer to short-term hospital	31995 (2.89)	30845 (2.89)	645 (3.09)	505 (2.81)	
Transfer to other facility: Includes Skilled Nursing Facility (SNF), Intermediate Care Facility (ICF), Another Type of Facility	468640 (42.37)	451890 (42.34)	9505 (45.49)	7245 (40.34)	
Home Health Care	156825 (14.18)	148725 (13.94)	3805 (18.21)	4295 (23.91)	
Against Medical Advice	10685 (0.97)	10480 (0.98)	90 (0.43)	115 (0.64)	
Died	43545 (3.94)	40545 (3.80)	1230 (5.89)	1770 (9.86)	
Discharged alive, destination unknown	250 (0.02)	215 (0.02)	20 (0.10)	15 (0.08)	

Table S4. Patient characteristics on admission, stratified by the most prevalent 5 types of cancer: respiratory cancers, prostate cancer, breast cancer, pancreatic cancer or colorectal cancer.

	**Respiratory Cancers**	**Prostate Cancer**	**Breast Cancer**	**Pancreatic Cancer**	**Colorectal Cancer**
n	9490	4960	3375	2640	2490
Age (years), median (IQR)	71 (63–79)	79 (71–85)	74 (67–83)	71 (63–77)	74 (65–82)
Length-of-stay (days), median (IQR)	4 (3–7)	3 (2–6)	4 (2–6)	4 (3–7)	4 (2–8)
Sex Female, *n* (%)	4855 (51.16)	0 (0.00)	3355 (99.41)	1320 (50.00)	1290 (51.81)
**Ethnicity**					
White	7050 (74.29)	3265 (65.83)	2375 (70.37)	1780 (67.42)	1615 (64.86)
Black	1155 (12.17)	935 (18.85)	505 (14.96)	435 (16.48)	415 (16.67)
Hispanic	445 (4.69)	325 (6.55)	205 (6.07)	170 (6.44)	215 (8.63)
Asian or Pacific Islander	340 (3.58)	100 (2.02)	65 (1.93)	85 (3.22)	85 (3.41)
Native American	15 (0.16)	15 (0.30)	<11*	<11*	20 (0.80)
Other	220 (2.32)	95 (1.92)	70 (2.07)	115 (4.36)	35 (1.41)
**ELIXHAUSER CO-MORBIDITIES**, *n* (%)					
Congestive Heart Failure	1215 (12.80)	765 (15.42)	480 (14.22)	270 (10.23)	460 (18.47)
Valvular Disease	880 (9.27)	590 (11.90)	350 (10.37)	260 (9.85)	265 (10.64)
Pulmonary Circulation Disease	620 (6.53)	50 (1.01)	50 (1.48)	335 (12.69)	110 (4.42)
Peripheral Vascular Disease	1255 (13.22)	545 (10.99)	340 (10.07)	195 (7.39)	230 (9.24)
Paralysis	1105 (11.64)	500 (10.08)	285 (8.44)	225 (8.52)	275 (11.04)
Other Neurological Disorders	70 (0.74)	45 (0.91)	20 (0.59)	15 (0.57)	45 (1.81)
Chronic Pulmonary Disease	3745 (39.46)	680 (13.71)	580 (17.19)	325 (12.31)	390 (15.66)
Diabetes (without chronic complications)	1370 (14.44)	835 (16.83)	580 (17.19)	700 (26.52)	440 (17.67)
Diabetes (with chronic complications)	890 (9.38)	740 (14.92)	445 (13.19)	360 (13.64)	400 (16.06)
Hypothyroidism	1090 (11.49)	415 (8.37)	675 (20.00)	325 (12.31)	355 (14.26)
Renal Failure	1180 (12.43)	1065 (21.47)	435 (12.89)	300 (11.36)	420 (16.87)
Liver Disease	175 (1.84)	85 (1.71)	40 (1.19)	80 (3.03)	85 (3.41)
Peptic Ulcer Disease	35 (0.37)	35 (0.71)	70 (2.07)	5 (0.19)	25 (1.00)
Acquired Immune Deficiency Syndrome	25 (0.26)	15 (0.30)	<11*	<11*	<11*
Lymphoma	85 (0.90)	15 (0.30)	25 (0.74)	20 (0.76)	30 (1.20)
Rheumatoid Arthritis / Collagen Vascular Disease	310 (3.27)	85 (1.71)	115 (3.41)	50 (1.89)	70 (2.81)
Coagulopathy	1170 (12.33)	340 (6.85)	210 (6.22)	500 (18.94)	230 (9.24)
Obesity	415 (4.37)	395 (7.96)	390 (11.56)	205 (7.77)	215 (8.63)
Weight loss	1270 (13.38)	290 (5.85)	225 (6.67)	420 (15.91)	300 (12.05)
Fluid and electrolyte disorders	2740 (28.87)	1150 (23.19)	910 (26.96)	885 (33.52)	750 (30.12)
Anaemia (chronic blood loss)	60 (0.63)	20 (0.40)	<11*	<11*	160 (6.43)
Anaemia (deficiency)	2400 (25.29)	1070 (21.57)	590 (17.48)	795 (30.11)	870 (34.94)
Alcohol abuse	365 (3.85)	180 (3.63)	65 (1.93)	80 (3.03)	100 (4.02)
Drug abuse	165 (1.74)	70 (1.41)	30 (0.89)	20 (0.76)	25 (1.00)
Psychoses	205 (2.16)	85 (1.71)	105 (3.11)	45 (1.70)	35 (1.41)
Depression	1070 (11.28)	435 (8.77)	475 (14.07)	320 (12.12)	255 (10.24)
Hypertension	6750 (71.13)	4230 (85.28)	2720 (80.59)	1870 (70.83)	1980 (79.52)
**OTHER CO-MORBIDITIES**, *n* (%)					
Sepsis	280 (2.95)	100 (2.02)	75 (2.22)	130 (4.92)	125 (5.02)
Liver Disease	215 (2.27)	100 (2.02)	55 (1.63)	115 (4.36)	100 (4.02)
Anaemia	3080 (32.46)	1215 (24.50)	790 (23.41)	975 (36.93)	1205 (48.39)

Dyslipidaemia	4355 (45.89)	2725 (54.94)	1580 (46.81)	1130 (42.80)	1160 (46.59)
Dementia	550 (5.80)	780 (15.73)	305 (9.04)	95 (3.60)	240 (9.64)
Smoking	2145 (22.60)	500 (10.08)	430 (12.74)	335 (12.69)	280 (11.24)
Parkinson Disease	70 (0.74)	125 (2.52)	35 (1.04)	<11*	15 (0.60)
Transient Ischaemic Attack	30 (0.32)	60 (1.21)	25 (0.74)	<11*	<11*
Rheumatic Heart Disease	270 (2.85)	175 (3.53)	140 (4.15)	60 (2.27)	75 (3.01)
Coronary Heart Disease	2960 (31.19)	1715 (34.58)	700 (20.74)	780 (29.55)	745 (29.92)
Heart Failure	1355 (14.28)	905 (18.25)	535 (15.85)	305 (11.55)	530 (21.29)
Pulmonary Embolism	575 (6.06)	45 (0.91)	40 (1.19)	325 (12.31)	100 (4.02)
Congenital Heart Disease	330 (3.48)	110 (2.22)	60 (1.78)	110 (4.17)	30 (1.20)
Pericarditis	<11*	<11*	<11*	<11*	<11*
Infectious Endocarditis	80 (0.84)	<11*	25 (0.74)	40 (1.52)	<11*
Atrial Fibrillation	2330 (24.55)	1595 (32.16)	975 (28.89)	535 (20.27)	710 (28.51)
Arrhythmias (other than Atrial Fibrillation)	730 (7.69)	545 (10.99)	285 (8.44)	165 (6.25)	190 (7.63)
Peripheral Vascular Disease	1270 (13.38)	565 (11.39)	355 (10.52)	205 (7.77)	230 (9.24)
Deep Venous Thrombosis	795 (8.38)	90 (1.81)	75 (2.22)	405 (15.34)	110 (4.42)
Gastrointestinal Bleeding	145 (1.53)	45 (0.91)	30 (0.89)	65 (2.46)	245 (9.84)
Pneumonia	1015 (10.70)	135 (2.72)	165 (4.89)	125 (4.73)	105 (4.22)
Chronic Lung Disease	3625 (38.20)	590 (11.90)	465 (13.78)	265 (10.04)	310 (12.45)
Chronic Obstructive Pulmonary Disease	3310 (34.88)	505 (10.18)	440 (13.04)	230 (8.71)	265 (10.64)
Ventricular Septal Defect	<11*	<11*	<11*	<11*	<11*
Atrial Septal Defect	315 (3.32)	110 (2.22)	60 (1.78)	105 (3.98)	30 (1.20)
Shock	100 (1.05)	15 (0.30)	15 (0.44)	20 (0.76)	15 (0.60)
Family history of cerebrovascular disease	235 (2.48)	120 (2.42)	90 (2.67)	50 (1.89)	95 (3.82)
Family history of heart disease	385 (4.06)	190 (3.83)	140 (4.15)	85 (3.22)	90 (3.61)
Previous cerebrovascular disease	1190 (12.54)	730 (14.72)	455 (13.48)	300 (11.36)	295 (11.85)
**PROCEDURES**, *n* (%)					
Thrombectomy	405 (4.27)	130 (2.62)	80 (2.37)	120 (4.55)	90 (3.61)
Thrombolysis	445 (4.69)	440 (8.87)	320 (9.48)	135 (5.11)	145 (5.82)
**OUTCOMES**, *n* (%)					
In-hospital mortality	965 (10.17)	185 (3.73)	190 (5.63)	280 (10.61)	155 (6.22)
Length-of-stay >4 days	4470 (47.10)	1850 (37.30)	1280 (37.93)	1295 (49.05)	1170 (46.99)
Routine Discharge	2010 (21.31)	1505 (30.47)	995 (29.57)	475 (18.06)	635 (25.76)
**OTHER CHARACTERISTICS**, *n* (%)					
**Year of admission**					
2015	935 (9.85)	530 (10.69)	345 (10.22)	310 (11.74)	280 (11.24)
2016	4265 (44.94)	2180 (43.95)	1275 (37.78)	1145 (43.37)	1035 (41.57)
2017	4290 (45.21)	2250 (45.36)	1755 (52.00)	1185 (44.89)	1175 (47.19)
**Hospital bedsize**					
Small	1225 (12.91)	820 (16.53)	550 (16.30)	395 (14.96)	410 (16.47)
Medium	2740 (28.87)	1445 (29.13)	1060 (31.41)	695 (26.33)	695 (27.91)
Large	5525 (58.22)	2695 (54.33)	1765 (52.30)	1550 (58.71)	1385 (55.62)
**Location/teaching status of hospital**					
Rural	540 (5.69)	335 (6.75)	325 (9.63)	150 (5.68)	175 (7.03)
Urban non-teaching	2130 (22.44)	1175 (23.69)	740 (21.93)	460 (17.42)	535 (21.49)
Urban teaching	6820 (71.87)	3450 (69.56)	2310 (68.44)	2030 (76.89)	1780 (71.49)
**Region of hospital**					
Northeast	1890 (19.92)	1070 (21.57)	680 (20.15)	625 (23.67)	405 (16.27)
Midwest	2180 (22.97)	1095 (22.08)	780 (23.11)	475 (17.99)	575 (23.09)
South	3765 (39.67)	1755 (35.38)	1285 (38.07)	1010 (38.26)	1080 (43.37)
West	1655 (17.44)	1040 (20.97)	630 (18.67)	530 (20.08)	430 (17.27)

**All Patient Refined DRG: Severity of Illness Subclass**					
Minor loss of function	130 (1.37)	280 (5.65)	165 (4.89)	20 (0.76)	50 (2.01)
Moderate loss of function	2120 (22.34)	2130 (42.94)	1415 (41.93)	590 (22.35)	780 (31.33)
Major loss of function	5015 (52.85)	2010 (40.52)	1410 (41.78)	1450 (54.92)	1170 (46.99)
Extreme loss of function	2225 (23.45)	540 (10.89)	385 (11.41)	580 (21.97)	490 (19.68)
**Disposition of the patient at discharge**					
Routine Discharge	2010 (21.18)	1505 (30.34)	995 (29.48)	475 (17.99)	635 (25.50)
Transfer to short-term hospital	285 (3.00)	180 (3.63)	40 (1.19)	70 (2.65)	45 (1.81)
Transfer to other facility: Includes Skilled Nursing Facility (SNF), Intermediate Care Facility (ICF), Another Type of Facility	3950 (41.62)	2275 (45.87)	1440 (42.67)	985 (37.31)	1130 (45.38)
Home Health Care	2220 (23.39)	795 (16.03)	700 (20.74)	820 (31.06)	500 (20.08)
Against Medical Advice	50 (0.53)	15 (0.30)	<11*	<11*	15 (0.60)
Died	965 (10.17)	185 (3.73)	190 (5.63)	280 (10.61)	155 (6.22)
Discharged alive, destination unknown	<11*	<11*	<11*	<11*	<11*

IQR – inter-quartile range; * cell sizes ≤10 were not reported in order to avoid patient re-identification, according to the HCUP guidelines [21]

Table S5. Results of multivariable logistic regressions evaluating the associations between non-metastatic and metastatic cancer and the odds of receiving thrombolysis or thrombectomy for acute ischaemic stroke.

	**Intravenous Thrombolysis**	**Endovascular Thrombectomy**
	**OR (95% CI)**	**OR (95% CI)**
No cancer	Reference	Reference
Non-metastatic cancer	**0.77 (0.66–0.90)**	**0.80 (0.63–0.9994)**
Metastatic cancer	**0.39 (0.32–0.47)**	0.87 (0.69–1.09)

Models adjusted for age, sex, ethnicity, Elixhauser co-morbidities (congestive heart failure, valvular disease, pulmonary circulatory disease, peripheral vascular disease, paralysis, other neurological disorders, chronic pulmonary disease, diabetes, hypothyroidism, renal failure, liver disease, peptic ulcer disease, acquired immune deficiency syndrome, rheumatoid arthritis, coagulopathy, obesity, weight loss, fluid and electrolyte disorders, anaemia, alcohol abuse, drug abuse, psychosis, depression and hypertension), previous history of cancer, haematological cancers, other co-morbidities (dyslipidaemia, smoking, Parkinson disease, coronary heart disease, all-cause bleeding, pulmonary embolism, deep venous thrombosis, atrial fibrillation, arrhythmias other than atrial fibrillation, pneumonia (incl. aspiration), shock, previous cerebrovascular disease), hospital bedsize, location & teaching status and other revascularisation therapy. Statistically significant differences ($p < 0.05$) highlighted in **bold**.
